# The Bunya Project: Protocol for a Mixed-Methods Approach to Developing a Culturally Informed Curriculum

**DOI:** 10.2196/39864

**Published:** 2023-05-18

**Authors:** Danielle Manton, Megan Williams, Andrew Hayen

**Affiliations:** 1 Girra Maa Indigenous Health Discipline School of Public Health University of Technology Sydney Sydney Australia; 2 School of Public Health University of Technology Sydney Sydney Australia

**Keywords:** Indigenous, university, curriculum, leadership, cocreation, self-determination

## Abstract

**Background:**

Indigenous peoples live across all continents, representing approximately 90 nations and cultures and 476 million people. There have long been clear statements about the rights of Indigenous peoples to self-determine services, policies, and resource allocations that affect our lives, particularly via the United Nations Declaration on the Rights of Indigenous Peoples. An area for urgent improvement is curricula that train the predominantly non-Indigenous health workforce about their responsibilities and that offer practical strategies to use when engaging with Indigenous peoples and issues.

**Objective:**

The Bunya Project is designed to advance Indigenous community-led teaching and evaluation of the embeddedness of strategies to achieve an Indigenous Graduate Attribute in Australia. The project centers the relationships with Aboriginal community services to lead education design relating to Indigenous peoples. The project aims to articulate community recommendations for university education in allied health in the usable format of digital stories to create culturally informed andragogy, curriculum, and assessment measures for use in teaching. It also aims to understand the impact of this work on student attitudes and knowledge about Indigenous peoples’ allied health needs.

**Methods:**

Multilayered project governance was established, along with a 2-stage process using mixed methods participatory action research and critical reflection, using the reflective cycle by Gibbs. The first stage, *preparing the soil*, used community engagement, drew on lived experience, encouraged critical self-reflection, embodied reciprocity, and demanded working collectively. The second stage, *planting the seed*, requires more critical self-reflection, the development of community data through interviews and focus group discussions, the development of resources with an academic working group and community participants, the implementation of those resources with student feedback, the analysis of the feedback from students and community members, and reflection.

**Results:**

The protocol for the first stage, *preparing the soil*, is complete. The results of the first stage are the relationships built and the trust earned and gained, and it has resulted in the development of the *planting the seed protocol*. As of February 2023, we have recruited 24 participants. We will analyze data shortly and expect to publish the results in 2024.

**Conclusions:**

The readiness of non-Indigenous staff to engage with Indigenous communities has not been ascertained by Universities Australia, nor can it be assured. Staff preparation and skills to support the curriculum, create a safe learning environment, and develop teaching and learning strategies to guide academics to recognize that how students learn is as important as the content students learn. This learning has broad implications and benefits for staff and students within their professional practice and for lifelong learning.

**International Registered Report Identifier (IRRID):**

DERR1-10.2196/39864

## Introduction

### Overview

There are 476 million Indigenous peoples worldwide, who belong to the world’s oldest continuing cultures and who have the right to self-determine health services to meet needs [1]. Health services are built upon and use the services of a predominantly tertiary-educated workforce. However, university health education contains little about Indigenous rights or knowledge [2]. Although immersion in cultural settings is recommended to stimulate transformation in knowledge and skills, current health curricula in Australia offer little toward this [3]. With few Indigenous staff at universities, responsibility lies with non-Indigenous staff, who are often underconfident in selecting and conveying information about Indigenous peoples [3]. Partnerships with Indigenous health organizations as experts in cultural models of care are essential; however, there are few examples in the literature to guide this [3]. An area for urgent improvement is curricula that train the predominantly non-Indigenous health workforce about their responsibilities and practical strategies they must use when engaging with Indigenous peoples and issues.

The Bunya Project is part of a participatory action research (PAR) project that aims to develop the skills and capabilities of academic staff to understand the principles and processes required to develop and embed Indigenous perspectives into the health care curriculum, ultimately influencing systemic change within the health care workforce.

This paper outlines a process for Indigenous community participation, showing how it is possible and important in developing culturally respectful engaging resources for students learning at university, across disciplines. The Bunya Project models processes and protocols for working with Indigenous peoples and community organizations that honor diverse Indigenous peoples’ knowledge with authenticity, humility, and reciprocity, as per cultural and ethical protocols. This paper contributes to the narrative as to how and why universities should partner with Indigenous communities and organizations; the value of the Bunya Project lies not only in sharing important knowledge held by Indigenous community organizations and members but also in demonstrating how to bring community into university life. This Bunya Project protocol may be useful for Indigenous peoples around the world, to adapt to local circumstances in partnership with community organizations and local teaching institutions.

The Bunya Project protocol outlined in this paper provides the principles and strategic knowledge used by a university education health faculty in Australia, driven by the Indigenous health team. The early stages of the protocol describe what could be called the “pre-research” stage—much of this preliminary work is taken for granted by the authors as “proper ways” or a respectful process, knowledge of which is gained through lived experiences and social expectations in a community context. However, what is obvious to us may not be obvious to non-Indigenous academics. Therefore, this paper outlines the principles and processes required to implement this protocol, based on the authors’ insider knowledge and lived experience as Indigenous peoples.

The Bunya Project is inspired by the tall Bunya tree of eastern Australia—*Araucaria bidwillii*—which grows slowly and, through its development, produces large pinecones that separate into edible nuts for current generations to be nourished by. It also spreads seeds for future generations to cultivate and grow. This Bunya Project provides a long-term, intergenerational, and unifying process for the voices of Indigenous peoples to be heard and shared in the allied health curriculum.

### Background

Aboriginal and Torres Strait Islander peoples are the Indigenous populations of the continent referred to as Australia. Aboriginal peoples are heterogenous, with >250 different language groups [4], each with their own protocols and ceremonies. The Torres Strait Islands comprise 5 island clusters, consisting of 38 inhabited islands and 113 islands in total. The islands lie between the tip of Queensland on the Australian continent and Papua New Guinea. Acknowledging this diversity is foundational to understanding and approaching teaching and learning within Indigenous contexts.

### Government Policy Context

In Australia, all national and state-based policies indicate that Aboriginal and Torres Strait Islander peoples have the right to cultures being at the center of decisions made, with full participation in decision-making [5,6] and its evaluation [7]. However, many of those currently in decision-making positions have had little education or support in understanding Australia’s history, cultures, or rights [8]. There are limited representative mechanisms for Aboriginal and Torres Strait Islander peoples to formally be included in decision-making, as there is an inherent power imbalance [9,10]. Those agreements made between governments and peak Aboriginal and Torres Strait Islander organizational bodies often have little resourcing and no evaluation, which limits both their ability to act and accountability [11].

### University Responsibilities and Realities

Current university education curricula produce graduates who often enter positions of power relevant to Indigenous peoples’ realization of rights. However, their education is severely lacking and does not produce graduates who are sufficiently well developed to bring about the types of urgent health system or health workforce changes needed to influence the drastic improvements required [3,12]. This is a common issue for Indigenous peoples globally [13].

The embedding of Indigenous peoples’ perspectives into mainstream university curriculum has been named for at least 2 decades in Australia [14,15] but only recently have structures been put in place by some universities [16,17] that help produce conditions for clear, evaluable actions. The peak body for Australian universities, Universities Australia, produced a strategy for 2017 to 2020 [18] and an Indigenous strategy for 2022 to 2025 [19], both of which make leadership statements but have not been wholly evaluated or updated for future developments.

Nonetheless, most Australian universities have committed to embedding Indigenous perspectives into their curriculum [20] and are seeking Indigenous peoples’ leadership [21]. A specific strategy has been introduction of an “Indigenous Graduate Attribute” (IGA), a statement of attainment that is expected of all graduates in relation to Indigenous peoples [22]. In the health context, the IGA aims to improve graduates’ knowledge about Indigenous peoples and issues and, where possible, provide experiences with Indigenous peoples, with the expectation that this will improve professional practice [23] and, ultimately, improve health outcomes for Indigenous peoples.

Unfortunately, there are few examples for universities about how to achieve the IGA. Universities have rarely met Indigenous staff targets [24], meaning that non-Indigenous people are largely responsible for developing and delivering curricula about Indigenous peoples and for driving the implementation and evaluation of the IGA. Perhaps in some recognition of this, Universities Australia recommends that tertiary institutions “build robust, respectful and collaborative partnerships between themselves and the Aboriginal and Torres Strait Islander communities that they serve” [14]. There are few practical strategies, scaffolding, or implementation guidance for university staff to create Indigenous community collaborations.

As an example of uninformed curriculum content, information about Indigenous peoples’ health and well-being often begins with a focus on the most easily available information in the public domain—the Australian Government’s Closing the Gap framework [25]. This framework has been designed to highlight inequity—the gap in processes and outcomes that Indigenous Australian peoples experience across many life domains compared with others in Australia [26]. In so doing, Closing the Gap powerfully reinforces negative stereotypes and assumptions about Indigenous Australian peoples, with no focus on strengths, local cultures, or worldviews about health. In the curriculum and in the classroom, this perpetuates stereotypes with a deficit lens [27] that is neither community driven nor reflective, entrenching in students’ minds the notion that Indigenous peoples do not have the expertise to enhance mainstream learning or contribute to developing their professional practice in providing health care.

The holistic collectivist notion of Indigenous Australian peoples’ health has much to offer to Western biomedicine in recognizing the whole person and their context: “Not just the physical wellbeing of an individual but refers to the social, emotional and cultural wellbeing of the whole community in which each individual is able to achieve their full potential as a human being, thereby bringing about the total wellbeing of their community. It is a whole-of-life view and includes the cyclical concept of life-death-life” [28].

To health educators who are versed in Western biomedicine, this holistic health concept seems complex and “too hard” [29]. Statistics used to measure and teach about health reinforce a power imbalance and dominance of the medical model [30]. With skilled, confident, and supported staff, more complex underlying contextual and environmental factors in health, including ongoing processes of colonization, systemic bias, and racism, are minimized [31]. Furthermore, the focus on Indigenous peoples’ ill-health does nothing to challenge students to understand the influence of their own cultural lens [32] or the future risks of continuing to render invisible and devalue Indigenous models of health care.

### Challenges in Allied Health Education

Allied health care professions are recognizably underdeveloped, particularly in relation to Indigenous peoples’ ways of knowing, being, and doing in the curriculum and clinical practice [3]. Allied health education has a long history of excluding Indigenous knowledge [3,33]. The Indigenous allied health workforce and student cohorts are small; there is an assumption that more Indigenous allied health practitioners and students are required, but there is little critical thought in supporting Indigenous holistic health care and how that might shape the future health workforce [34]. There are clear gaps in research about allied health care needs of Indigenous peoples, and there is no attention to minority populations of Indigenous peoples such as those in prisons and those with disability or multiple, compounding issues. This limits the scope of exemplars available to inform curriculum. These gaps persist despite allied health professional education having specific accreditation requirements, often specific to the discipline and with mandatory renewal [35]. Furthermore, for example, the Australian Physiotherapy Council has strengthened its accreditation requirements to achieve more Indigenous student enrollments and include cultural safety in the curriculum [36], reinforced by changes to the National Safety and Quality Health Service Standards [37]. The Indigenous Allied Health Association provides some resources to guide health professionals and support students [38]; however, the implementation and use of available resources vary across institutions [39].

### Expertise About Indigenous Health Does Not Lie in Universities

Statements implicating universities in perpetuating the disadvantage of Indigenous peoples’ experience have recently been met with mixed responses in Australia, including denial and confusion [39,40]. The cultural load that Indigenous Australian staff experience in mainstream organizations—the additional expectations on Indigenous staff to fulfill tasks in accordance with position descriptions and educate others about Indigenous cultures [41]—is beginning to be aired. However, the experience of the relatively small number of Indigenous staff at universities is seldom acknowledged—having to both cope with stereotypes and exclusion of Indigenous knowledge and having to rectify the curriculum to meet accreditation while being understaffed [42]. University employment is not sustainable for Indigenous staff without the support of Indigenous community—personally or to help shape curriculum. Expertise about Indigenous health does not lie in universities. It is in the Indigenous community, which generally has few university graduates but has valuable expertise, cultural protocols, and appropriate positioning to identify and discuss key issues affecting their community and successful solutions.

### Expertise of Indigenous Communities and Organizations

In contrast, mounting evidence exists for the success of Aboriginal community–controlled health organizations. The networked organizations (>140) across Australia have demonstrated leadership since the 1970s in effective and efficient holistic health care [43]. They address the social determinants of health [31] and recognize their successes emanating from continued cultural connections within Indigenous communities [44]. They carry across generations the Indigenous knowledge that has “multiple and interconnected discourses, social practices and knowledge technologies” [45] and they showcase Indigenous voices that carry within them the shared and lived experience of families and communities, beyond individuals [46]. Aboriginal community–controlled health organizations are now one of the largest workforces of Indigenous Australian peoples [47]; they demonstrate many strengths in models of care, staff, support, retention, and career progression [48] and are important leaders for any new curriculum development.

### Project Context

Bunya has been designed as a central strategy for a university faculty (the University of Technology Sydney [UTS] Faculty of Health) and a graduate health school (the UTS Graduate School of Health) to support students to achieve IGA and to deliver curriculum for students embarking on a career in allied health care, specifically pharmacy, physiotherapy, clinical psychology, orthoptics, genetic counseling, and speech pathology. Bunya explores both how curriculum development processes can include Indigenous communities and Indigenous community members’ experiences of allied health care.

The idea of seeking to connect a university health faculty with Indigenous communities to develop curriculum and to research the process and outcomes is very well supported by Australia’s peak body for health and medical research, the National Health and Medical Research Council (NHMRC). The NHMRC guidelines, *Ethical conduct in research with Aboriginal and Torres Strait Islander Peoples and communities* and *Keeping research on track II,* were developed to promote health, prevent harm, and encourage best practice [49,50]. The NHMRC has also developed these specific guiding principles for conducting studies with Aboriginal and Torres Strait Islander peoples [49,50]. Historically, studies conducted in Aboriginal and Torres Strait Islander communities were extremely negative [50]. Previous studies conducted in Indigenous communities were, in some cases, physically barbaric [51]. Studies were designed to disempower, oppress, and silence Aboriginal and Torres Strait Islander peoples [52]. It is only in recent years that studies have been designed to be of benefit to Aboriginal and Torres Strait Islander peoples [53]. Aboriginal and Torres Strait Islander peoples are described as the most overresearched population in the world [54]. Processes have been put in place by governing research bodies such as the NHMRC and the Aboriginal Health and Medical Research Council of New South Wales. Both guidelines, *Ethical conduct in research with Aboriginal and Torres Strait Islander Peoples and communities* and *Keeping research on track II* [49,50], have been instrumental in the design and development of the Bunya Project and this protocol.

We have divided the Bunya Project into 2 stages—preparing the soil and planting the seed. Owing to the nature of PAR, it is impossible to separate protocols and learnings as clearly as in papers describing other forms of research, such as a clinical trial. The learnings from the *preparing the soil* stage influence the protocol developed in the *planting the seed* stage. As such, this paper will present some of the findings from the first stage, and a subsequent paper will elucidate them more clearly. The paper was written at the time of the transition from the first stage to the second stage.

### Theoretical Frameworks

We have taken a PAR approach [55]. PAR is a qualitative research methodology that relies on the participation of the researcher and the participants to collaboratively gather and interpret data and findings throughout the project. The process of PAR is flexible and responsive to be able to adapt to the direction of the project, rather than being dictated or set from the beginning. PAR is a recognized Indigenous research methodology [56] designed to center and amplify Indigenous voices and self-determination [57], which is an underpinning value of the Bunya Project. PAR reduces power inequity and helps to ensure reciprocity.

PAR also reflects the authors’ lived experiences of working with the community and as educators in the university context.

PAR prioritizes researchers and participants forming partnerships to identify issues of local importance and determine ways to understand the issues and strategies for action [58,59]. Furthermore, PAR methodology is used to understand complexities and influence change [55]. This resonates with the research team, given that Indigenous peoples have always implemented participatory principles within community governance through collective consultation and collective action [57]. PAR celebrates the complexity related to multiple contexts, including unique community settings and university education settings [60], with the ability to be agile to adapt to changing settings, participants, and ways of understanding [60].

In developing and articulating the preliminary process and protocol, the reflective cycle principles by Gibbs [61] were applied in a critical self-reflection context. The reflective framework principles by Gibbs also guided the authors to unpack informal conversations within the community engagement process, ensuring that the community remained central to the process and principles articulated [62]. Therefore, no data have been collected from student participants or will be reported here from external research associated with the Bunya Project. This paper presents critical reflection data from the research team to inform the project and its stakeholders. In evaluating Bunya, these are the foundational aspects required to build and replicate the project.

## Methods

### Ethics Approval

The Bunya Project has been designed in accordance with NHMRC ethics guidelines regarding studies being conducted with Aboriginal and Torres Strait Islander peoples [49,50].

The Bunya Project has been approved by both the University of Technology Sydney Human Research Ethics Committee (ETH18-2618) and the Aboriginal Health and Medical Research Council of New South Wales Human Research Ethics Committee (1451/18).

### Preparing the Soil

#### Overview

To develop the Bunya Project and method, the authors took into account cultural ways of working, along with community protocols and expectations. Overall, 5 steps were necessary to “prepare the soil” for the Bunya Project process. The five steps are as follows:

1. Community engagement—developing relationships to ensure that the project is of benefit to the community and responsive to the community objectives
2. Lived experience—humility in recognizing the influence of our own lived experience on our worldviews and interactions
3. Critical self-reflection—about our own perspectives and the influence these perspectives and experiences have on the formulation of ideas, conduct, and expectations
4. Reciprocity—which ensures that the benefit is mutual and the communities are meeting their objectives, not what the researcher thinks is of benefit to them
5. Working collectively—which creates the space for all participants to belong and feel empowered as an important part of the team, process, and outcomes achieved

**Figure 1:**
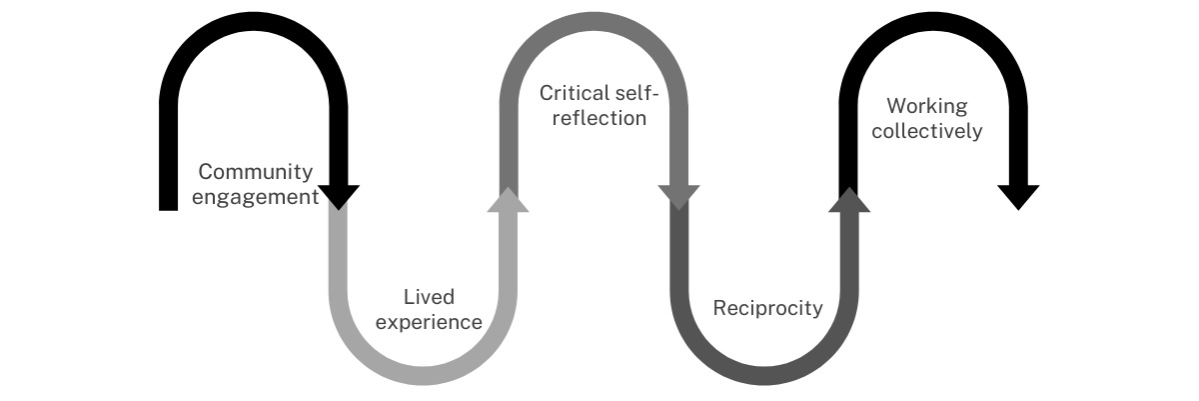
Preparing the soil in the Bunya Project.

Figure 1 shows these steps but note that they are fluid and may be repeated in part or whole throughout the process.

#### Community Engagement

We identified four priority groups to engage with:

1. Indigenous community organizations
2. Indigenous community members and health care service users
3. University staff
4. University students

Invitations for group 1, Indigenous community organizations, to participate in the Bunya Project were circulated through networks and social media to encourage self-nomination. The research team visited community organizations and attended community events, circulating information and responding to queries. In total, 6 organizations signed up as Bunya Project collaborators. During the COVID-19 pandemic, this was reduced to 5 owing to travel distance restrictions and then to 4 because 1 organization ceased operating. A formal agreement was negotiated with each organization about their participation, including supporting curriculum development, assisting with the recruitment of Indigenous individuals who are willing to participate in data collection, and providing mentoring and peer support during the Bunya Project.

The Indigenous community organizations invited community members and health care service users (group-2 participants) to participate either directly or through social media. Information about the Bunya Project was circulated within each organization. Participants then self-nominated to participate. Participation includes interviews to collect data pertaining to insight into their own health care needs and strengths within their community influencing educational opportunities for allied health education. A total of 24 Indigenous community members and health care users were recruited to be interviewed.

Group-3 participants, university staff, were recruited through self-nomination.

University students (group-4 participants) will be invited to participate in the Bunya Project via the unit coordinator or class tutors. Participation is voluntary. Students who self-nominate will be required to complete premethod and postmethod surveys; we are aiming to survey most participants (n=169, 75.1%), with the total being 225 students.

The Bunya Project encourages connectedness wherever possible among project participant groups.

#### Lived Experience

Lived experience recognizes the cultural, spiritual, physical, emotional, and mental experiences; circumstances; beliefs; and worldviews of a person, including the well-being of the individual, family, or community. A lived experience recognizes the ongoing impact of colonization experienced by Aboriginal and Torres Strait Islander individuals, families, and communities. Lived experience also recognizes specific events that have an impact on the social, emotional, and cultural well-being of Aboriginal and Torres Strait Islander peoples [63]. Lived experience as an Aboriginal person is complex, with many layers; is difficult to define but understood and recognized within the community and shared commonality; and does not need to be defined but is reflected through respecting protocols within the community [64]. Lived experience is an extremely valuable teaching tool, as students connect with shared lived experiences and the opportunity to learn from experiences that differ from their own. Lived experience also ensures that the teaching is authentic and relevant in a real-world context [65].

#### Critical Self-reflection

Critical self-reflection resulted in the identification of clear guiding principles for the conduct of the project. These guiding principles also reflect general cultural expectations when working with community and Indigenous knowledge. These principles are a guide for consideration in best practices for community engagement and leadership in teaching and learning and Indigenous research cultural protocols. As an Indigenous peoples–led research team, we cannot differentiate cultural protocols from research protocols; Indigenous culture informs knowledge production and the use of knowledge in research and practice.

#### Reciprocity

The research team actively participates in the Indigenous community organizations’ business, supporting initiatives and events; contributing to committees; contributing skills, knowledge, and expertise; and providing access to university resources and networks.

The project continues to progress with mutual understanding of and agreement on key activities.

#### Working Collectively

Although the leadership of the Bunya Project rests with the Indigenous health academic unit within the faculty, the leadership has a clear desire to work collectively. The heads of each of the 6 postgraduate health disciplines were invited to nominate a staff member to join an academic working group fondly termed “the Bunya Nuts.” These representatives provide discipline-specific expertise and guide curriculum development to ensure academic rigor and alignment with the curriculum that is already established.

The Bunya Nuts developed the terms of reference collectively; members are responsible for liaising within their discipline, inviting feedback, distributing community-developed video teaching resources, and facilitating student survey participation.

### Objectives

The Bunya Nuts also selected the following objectives for its mixed methods PAR project:

1. Develop relationships with Aboriginal community services to support the project and understand community recommendations for university education in allied health
2. Conduct interviews and collect digital stories from Aboriginal and Torres Strait Islander peoples, to understand “what are their experiences, perspectives, knowledges and aspirations for their own health and wellbeing and in relation to allied healthcare?”
3. Create culturally informed andragogy, curriculum, and assessment measures for use among teaching staff and students
4. Implement an evaluation framework to understand the effects on student attitudes and knowledge about Indigenous peoples’ allied health needs and aspirations and the authenticity, relevance, and accessibility of the curriculum for staff
5. Continue to develop culturally informed teaching and learning strategies

## Results

### Planting the Seed

#### Overview

This section discusses the results of the “preparing the soil” process of the Bunya Project. The “preparing the soil” process resulted in key insights for the “planting the seed” stage including how future data are to be collected and the process and methodology for replication within the reader’s own context.

**Figure 2:**
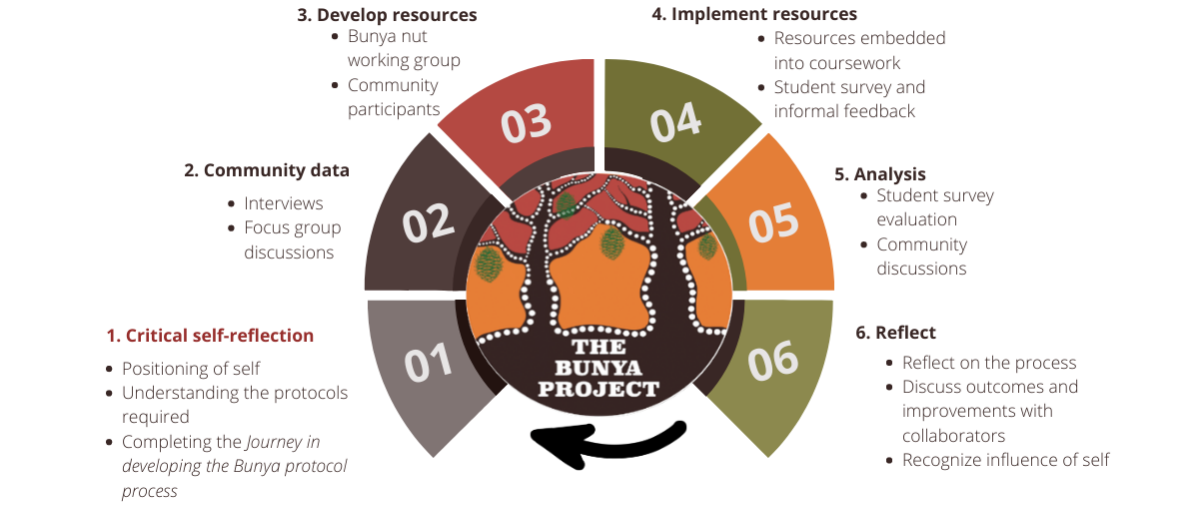
Planting the seed—Bunya Project protocol phases.

As outlined in this paper, the Bunya Project uses PAR cycles to gather information, develop curriculum, use it, and evaluate it from multiple perspectives. As a PAR project, the “planting the seed” stage of Bunya itself has 6 stages, each stage influencing the next in a continuous cycle, as illustrated in Figure 2.

The “planting the seed” process begins with critical self-reflection positioning by those guiding the project, their bias and influence, and ensuring that they have undertaken deliberate preparation to uphold the authenticity of the project. Phase 1 also incorporates the process in “preparing the soil” illustrated in Figure 1 previously. This involves recognizing their position, influence, and power and identifying the strategies and protocols required to privilege Indigenous voices and leadership and honor local protocols and expectations. Following this is community engagement and data collection; the data collected in each phase influence the following phases. Community data generate the content for the development and embedding of resources, and the resources activate thinking for the collaborative analysis of the survey and discussion. This is followed by a collaborative reflection about the process, improvements, and adjustments, and the cycle then commences again, with critical self-reflection of the person guiding the project.

As Figure 2 shows, Bunya is a PAR project because the overall community participation is central to all elements of the project. Phase 2 involves qualitative data collection, and phase 3 involves filming and editing the new teaching and learning resources and planning where in the curriculum they will be most effectively used, in collaboration with Bunya Nuts and community participants. Phase 4 involves using the resources and collecting a baseline survey from students and staff, and phase 5 involves the follow-up survey to help understand change and analysis of data. Phase 6 involves reflection with Bunya Nuts and community participants. PAR defines Bunya because it relies on community engagement throughout [55,58,61,66].

#### Critical Self-reflection

Critical self-reflection is again consciously used in the “planting the seed” stage of the Bunya Project, using the reflective cycle by Gibbs [61] to understand the experience, identify thoughts and feelings through the process, and recognize the importance of continuous improvement and the role of the researcher in modeling those processes as best practice. Undertaking this process at this point in the protocol provides the opportunity to highlight areas that may need to be revisited or unpacked further, in collaboration with the community involved.

#### Community Data

##### Focus Groups Discussions

In the future, focus group discussions will be conducted with Indigenous organization representatives. Focus group discussions encourage knowledge-producing dialogue in a group context. They allow the researcher to be part of the discussion [67]. This aligns with Indigenous ways of yarning and kapati, which encourages Indigenous peoples to talk freely about their experiences, thoughts, and ideas [68]. Furthermore, focus group discussions allow for broad discussion and framing and focusing questions and concepts to inform subsequent research phases, in this case, to inform semistructured interviews.

Focus group discussions will be semistructured, with guiding questions developed by the Bunya Nuts, and will be 30 to 60 minutes in duration.

The groups will have approximately 6 participants. Topics will range from health care knowledge and experiences to identifying needs and strengths within their community to influence the educational opportunities for tertiary education.

The focus group discussions will be audio recorded with written consent from all participants. Focus group participants will receive light refreshments in reciprocity of their time. All data collected during the focus groups will be deidentified.

##### Interviews

Interviews are designed to occur as a social interaction while sharing a cup of tea, known as kapati time, and create a safe space that facilitates yarning and storytelling [68]. Interviews stimulate knowledge sharing through storytelling and yarning [69]; storytelling is an important way of sharing information in Aboriginal and Torres Strait Islander cultures [67]. The interview structure will follow a line of questioning arising from the Bunya Nuts and the focus group discussions; however, this structure is intertwined with the informality of conversation and storytelling about experiences pertaining to the question.

The interviews will be video recorded, with individual consent obtained from each participant before participation. Videos—digital stories—are a way of clearly displaying that the individual holds ownership over their knowledge, culture, and experiences and tells their story themselves, rather than having someone interpret their experience. Videos will then be edited for use, and written final approval will be obtained from the participant before the videos are implemented as internal teaching resources. Videos remain as the property of the participant and are not permitted to be changed or altered in any way unless additional consent is sought from the participant. The 24 interview participants will receive a gift card worth Aus $20 (US $13.23) as compensation for their participation.

Overall, 6 interviews will be conducted at each of the 4 locations.

##### Premethod and Postmethod Surveys for Staff and Students

The survey chosen to be used for the Bunya Project was designed as a university-wide instrument by the Centre for the Advancement of Indigenous Knowledges [8]. It measures students’ attitudes, knowledge, and learning experiences pertaining to Indigenous ways of knowing, being, and doing. The premethod and postmethod survey measures whether the teaching and learning resources and strategies influenced change in staff and students’ attitudes.

The survey assessment framework is based on 5 overarching themes that are aimed to critically engage with teaching and learning content related to the university’s IGA. The overarching themes are as follows:

1. Knowledge
2. Critical reflectivity
3. Indigenous voice
4. Indigenous community engagement
5. Applied Indigenous knowledge [70]

The students will be provided with the survey through the survey platform. The Bunya Nuts representative for the discipline will upload the link to the learning management system for the subject. The link will be accompanied by an announcement to encourage student participation. The survey will remain open for at least 3 weeks, and students will be encouraged to complete it.

#### Develop Resources

The development of the resources begins with the Bunya Nuts working group to identify topics within the curriculum for each discipline. These topics will be discussed in focus group discussions with Aboriginal community–controlled health services, identifying the specific learning or information that the group determines as important for the students to know, understand, and apply within their professional practice. On the basis of the results of the discussions, one-on-one interviews will be conducted and video or audio recorded (according to participant preference). The interview questions and topics will be directly linked to the outcome of the focus group discussion, ensuring Indigenous self-determination throughout the process. The clips will be edited into short clips and reviewed by the participants to ensure that the message remained as intended.

#### Implement Resources

The resources developed will be made available to the school teaching staff within the various disciplines. The resources will be uploaded to the learning management system. Staff will be invited by the project team to access the resources. The project team will have control over who can access the resources and analytics to track where the resources are implemented within the learning management system, to ensure that the resources are only implemented in accordance with the ethical guidelines. The teaching staff will copy the resources into various points within their subjects to ensure that an Indigenous voice is present. The implementation of the resources will be accompanied by support processes outside this project, including teaching principles, an andragogy framework, and professional development opportunities for the staff. The project team recognizes the importance of not only what is taught (the content) but also how it is taught (the andragogy).

#### Analysis

The analysis of the data will be undertaken by the research team, in collaboration with the community partners and the Bunya Nuts. The andragogy framework guides the analysis, centering Indigenous knowledge and learning methodology within the context of adult education. Specific analysis will be conducted for each data set—the focus group discussions, interviews, and surveys.

Interviews will be transcribed and entered into NVivo (QSR International) [71] to be coded, with themes arising from the data. Codes will be compared, condensed, and organized into high-level themes to be communicated [72]. Focus group discussions will be managed similar to interviews, with the inclusion of group analysis [73]. Drafts and ideas about the data will be shared with the participating community organizations for their feedback. Furthermore, the qualitative interviews will be transformed and edited into digital stories, to be given to each participant and produce teaching resources for the disciplines. We will also draw on facilitator’s written notes made during the focus group discussions.

The Centre for the Advancement of Indigenous Knowledges’ processes for the analysis of data will be followed. The results of the validated survey will be analyzed to measure the impact of the authentic community-controlled teaching resources on the staff and students’ attitudes, knowledge, and learning experiences in relation to Indigenous perspectives within their professional learnings. The quantitative data will be carefully cleaned, with frequencies identified using SPSS [74] to identify overall patterns, including across the 5 basic themes the instrument focuses on. Other basic statistical data analysis will occur including averages and partial correlation for associations with student outcomes.

Drafts and initial thoughts will again be shared with the community organizations to provide feedback regarding the progress and impact of the resources developed. The community guidance will influence modifications in the teaching resources for future implementation, beyond the scope of this project.

### Current Status

The protocol for the first stage, *preparing the soil*, is complete. The results of the first stage are the relationships built and the trust earned and gained, and it has resulted in the development of the *planting the seed protocol*. As of February 2023, we have recruited 24 participants. We will analyze data shortly and expect to publish the results in 2024.

## Discussion

### Summary

Through the “preparing the soil” stage of the PAR, the “planting the seed” Bunya Project protocol emerged, as depicted in Figure 2. This represents key findings, the importance of critical self-reflections in Indigenous health education and PAR, the importance of holding space in curriculum development and design to privilege Indigenous voices through the engagement of community data, collective development of resources, implementing resources in a culturally safe learning space, the analysis of the learning content and process, and reflection to ensure continuous improvement of the process and educators.

PAR is an empowering methodology that privileges local knowledge and lived experiences throughout every facet of the project [56,57]—the direction, implementation, analyses, and outcome. The Indigenous perspectives embedded within the curriculum are based on establishing relationships with the local community that are reciprocal and collaborative with Indigenous leadership and the university through the Bunya Nuts academic working group. The collaboration between the Bunya Nuts and the community is instrumental in the creation of new knowledge as this collaboration develops the capacity of both parties learning from each other through action [57,60,69]. The collective action involves creating videos and resources that honor and reflect the intended meaning and context from community to academia and from academia to community. This action extends to influence change in allied health care through educating and building a culturally responsive workforce. Bunya’s andragogic framework is designed to reinforce the content, collaboration, and leadership of the community as the authoritative voice and knowledge holders [67].

This study encourages universities to develop partnerships with Indigenous community organizations in curriculum development. Establishing and maintaining authentic partnerships with Indigenous community organizations is important for the development of the future workforce and provides essential knowledge and skills for all graduates. Academic staff and institutions must move past an “othering” mindset, including the notion that developing relationships with Indigenous community organizations and including Indigenous perspectives in the curriculum is the sole responsibility of Indigenous academic staff. The reality is that Indigenous staff numbers in universities are low, globally. Embedding Indigenous perspectives into the curriculum is the responsibility and privilege of all the academic staff. It is not possible to create a checklist or a descriptive “how to” guide, as teaching and learning must be grounded and relevant to the local context. This paper outlines the guiding principles developed from the lived experience and reflectivity of the authors as community members and educators.

In Australia, government policy and legislative requirements have been introduced to ensure that curricula and workplaces implement training and professional development opportunities for students and staff, so that they can develop and apply knowledge and skills that create conditions for cultural safety and culturally responsive health care. Authentic partnerships with community organizations are an obvious place to start. Students can then commence their career with a strong foundation by understanding how to work authentically with Indigenous organizations and peoples. This paper outlines how to approach and achieve this outcome.

As this paper also shows, partnerships require reciprocal benefit and authenticity, creating the opportunity for universities to positively and authentically work with Indigenous community organizations to share knowledge skills and resources that will benefit not only the university and the students but also the community organizations and their staff and clients.

### Comparisons With Previous Studies

The Bunya Project protocol unifies elements identified by past studies to form a comprehensive approach to embedding Indigenous perspectives within higher education with community engagement and leaders, andragogical framework, staff accountability, and student evaluation.

The growing momentum to include Indigenous perspectives into higher education and preparing graduates to work effectively with Indigenous populations is positive. However, current implementation processes in Australia lack accountability [63], evidence, and evaluation [8].

It has also been identified that Indigenous peoples cannot be solely responsible for all learning and teaching in academia. Indigenous and non-Indigenous academic staff must work together to implement the curriculum, using culturally safe [75] and relevant teaching and learning strategies [68,76,77]. This extends to the professional context whereby previous studies identified the importance of Aboriginal health workers and mainstream allied health workers working together within their professional context [75].

In accordance with United Nations Declaration on the Rights of Indigenous Peoples, Indigenous peoples have the right to self-determine health care and education [78]; therefore, they must be leaders in designing and informing curriculum, ensuring that learning is *with* Indigenous peoples and not *about* Indigenous peoples. This is demonstrated in various ways through previous studies, by applying and building on knowledge and experience with Indigenous peoples [79] to inform curriculum, through local Indigenous ways of knowing and learning in the classroom, and through community-engaged learning.

Previous studies in the area have identified the importance of the local context, of working with a single community—this does not reflect the diversity within Indigenous populations in Australia. The Bunya Project protocol extends this to include leadership from a diversity of communities.

The Bunya Project protocol also challenges educators to push past the concept of pedagogy to ensure that teaching and learning are receptive to the adult learner [80].

Transformational learning is central to this space and was identified throughout previous studies [32]. The San’yas Indigenous Cultural Safety Training in Canada is implemented as an educational intervention to promote transformational change in health systems, policies, and practice [81]. The Bunya Project protocol incorporates and builds on the importance of transformational learning and recognizing the position of self by all involved, including community participants, academic staff, and students.

### Limitations

COVID-19 affected the project, as lockdowns restrict interactions. Even during times of eased restrictions, communities were very cautious to engage owing to the risk posed by COVID-19 and have cancelled events or adapted to web-based events. However, the pandemic provided another opportunity to highlight Indigenous excellence as the overall response to the pandemic, including public health messaging and responses, and the rapid adaptability to web-based connectedness has been exemplary.

The Bunya Project was developed in 1 university and 6 communities, which does not reflect the diversity of Aboriginal peoples or Indigenous peoples globally.

### Conclusions

This study reinforces the necessity for universities to be active in engaging with Indigenous communities to influence education content and, consequently, outcomes. It is not the responsibility of the few Indigenous staff to champion this; the implication is that the majority must authentically engage with the community and privilege their leadership and expertise within teaching and learning design, delivery, and evaluation.

The value and implication of the Bunya Project lies in its demonstration of how universities resource staff to engage with community organizations and members within the university setting. If working well with Indigenous peoples requires the readiness of non-Indigenous staff and students, universities need to provide staff with cultural capability training, facilitating opportunities to learn. This Bunya Project protocol and its “preparing the soil” and “planting the seeds” stages model a process for best practice and authentic engagement that creates space, respect, and recognition of the value and privilege to learn from Indigenous leadership and expertise.

PAR of the Bunya Project is working to evolve university education through the quality delivery of an IGA that prepares students to be informed professionals throughout their careers and to contribute to creating conditions for Indigenous peoples to experience cultural safety; this is a lifelong learning journey. The intended learnings from the Bunya Project are expected to extend beyond university through continuing personal and professional development and application throughout the individual’s career.

## Abbreviations

IGA: Indigenous Graduate Attribute
NHMRC: National Health and Medical Research Council
PAR: participatory action research
UTS: University of Technology Sydney

## Data Availability

Data will be made available; however, they have not been collected yet.
